# Awareness and Uptake of HIV Preexposure Prophylaxis and Postexposure Prophylaxis Among College Students With Sexual Experiences: Institutional-Based Cross-Sectional Study

**DOI:** 10.2196/63211

**Published:** 2024-11-06

**Authors:** Junfang Xu, Ke Xu, Omar Juma, Xingliang Zhang, Zhujun Lian

**Affiliations:** 1 School of Public Health, the Second Affiliated Hospital, Zhejiang University School of Medicine, Hangzhou, China; 2 Department of AIDS/STDs Control and Prevention, Hangzhou Center for Disease Control and Prevention (Hangzhou Health Supervision Institution), Hangzhou, China; 3 Ifakara Health Institute Bagamoyo Office, Bagamoyo, United Republic of Tanzania; 4 Medical Office, Lishui People's Hospital, 15 Dazhong Road, Liandu District, Lishui, 323000, China, 86 13588819303

**Keywords:** HIV, preexposure prophylaxis, postexposure prophylaxis, college students, China

## Abstract

**Background:**

Evidence has shown that HIV prevalence among young people, especially college students, has increased disproportionately. Preexposure prophylaxis (PrEP) and postexposure prophylaxis (PEP) are two of the most effective ways to prevent HIV, which are vital for college students with sexual experiences who have sexual risks.

**Objective:**

To provide evidence for effective intervention to reduce the risk of HIV infection among young students, this study aimed to analyze the awareness and uptake of HIV PrEP and PEP among college students with sexual experiences.

**Methods:**

An institutional-based cross-sectional study design was used to collect data through an electronic questionnaire from college students in 5 colleges located in Zhejiang Province. A total of 21,962 college students were investigated, of which 2605 students with sexual experiences were included in the data analysis with the following information collected: sociodemographic characteristics, awareness and uptake of HIV PrEP and PEP, sexual behaviors, and HIV tests. Binary logistic regression analysis was used to explore the factors on seeking PrEP and PEP.

**Results:**

The average age of college students with sexual experiences was 21.25 (SD 2.75) years. Overall, 61.4% (n=1600) of the participants were aware of PrEP, and 53.0% (n=1380) of them were aware of PEP. Moreover, 5.6% (n=146) of them have sought PrEP or/and PEP, and 89.1% (n=2321) have not sought PrEP or PEP. College students who had more than 6 sexual partners, have always had unprotected sex, have subjective perceived risk behavior, and undergo HIV testing were more likely to seek PrEP or/and PEP. The main ways for the participants to learn PrEP and PEP were through school clubs, the internet, and the Centers for Disease Control and Prevention. Moreover, senior students and those who had not undergone an HIV test had a lower likelihood of seeking PrEP and PEP. College students who did not have risky sexual behaviors (odds ratio 0.468, *P*=.004) and homosexual students (odds ratio 0.318, *P*=.03) were more likely not to seek PEP.

**Conclusions:**

College students with sexual experiences rarely seek PrEP and PEP, with a relatively low awareness of PrEP and PEP. It is very important to increase the knowledge and uptake of PrEP and PEP by educational and behavioral interventions among young students at risk for HIV infection.

## Introduction

The UNAIDS (Joint United Nations Programme on AIDS) reported that 39.9 million people were living with HIV globally, and there were still an estimated 1.3 million new HIV infections and 630,000 AIDS-related deaths in 2023 [1]. Over 3000 new infections have been reported among young students in China, and more than 80% of students were infected through male homosexual transmission in 2018 [2]. The number of newly diagnosed college students has seen an annual growth rate ranging from 30% to 50% in China [3].

A cross-sectional study indicates that the incidence of sexual experiences among college students in mainland China has reached 18.7%, and with an uptrend, the incidence of sexual behavior among male college students was 27.0%, and that among female college students was 13.9% [4]. With the development of the social economy, the social environment is becoming more likely to be sexually permissive [5]. College students with different backgrounds and sexual orientations meet and live together without parental and administrative prohibitions [5]. In addition, with the popularity of the internet and the rise of mobile social software, college students can be exposed to sexual content from various channels and find it more convenient to access various potential sexual partners. Although more factors facilitate sexual behaviors, the level of risk awareness was poor. It has been shown that 60.61% of sexually active students had used a condom for every sexual encounter during a given year and the incidence of commercial sex among college students was 9% and that of male gay sex was 7% [6,7]. These college students with sexual experiences are more likely to be infected with HIV. Preexposure prophylaxis (PrEP) and postexposure prophylaxis (PEP) are two of the most effective ways to prevent HIV infection and save lives.

PrEP includes medications (pills or shots) that reduce one’s chances of contracting HIV. PrEP is for adults and adolescents without HIV who may be exposed to HIV through sex or injection drug use [8]. When used as prescribed, PrEP reduces the risk of contracting HIV from sex by about 99% and injection drug use by at least 74% [9]. PEP includes medications that prevent HIV after a possible exposure. PEP must be initiated within 72 hours (3 days) after a recent possible exposure to HIV [10]. During the past few decades, the provision of HIV PEP has been extended to nonoccupational exposures, including unprotected sexual exposure, injecting drug use, and exposure following sexual assault [11]. A retrospective case-control study demonstrated that the odds of HIV infection among health care workers who took PEP after exposure were reduced by approximately 81% [12]. Current research on HIV PrEP and PEP mainly focuses on the efficacy, willingness to use, drug compliance among drug users, sex workers, men who have sex with men, and health care workers. The research on awareness and uptake of HIV PrEP and PEP among college students who had sexual experiences was limited. Given this background, we aimed to analyze the awareness and uptake of HIV PrEP and PEP among college students with sexual experiences. Our findings could provide evidence for effective intervention to reduce the risk of HIV infection among young students.

## Methods

### Participants

We used an institutional-based cross-sectional study design to collect data from colleges located in Hangzhou, Zhejiang Province, China. From November 2022 to May 2023, a questionnaire survey about HIV prevention was conducted. Adopting a phased cluster sampling method, different types of schools were sampled on the basis of their distribution and settings. Students in various grades from 5 universities in Hangzhou (including comprehensive, science, polytechnics, and medical and vocational colleges) were invited to participate in the survey based on an electronic questionnaire. Public health or HIV prevention staff at schools or school hospitals provided the QR code to the participants, who scanned the QR code with their mobile phone and filled out the electronic questionnaire. A total of 21,962 college students completed the questionnaire, and among them, 2605 college students with sexual experiences were included into our analysis.

### Data Collection and Measurement

The following information was collected: sociodemographics (ie, age, gender, grade, gender of the sexual partner, and sexual orientation), awareness and uptake of HIV PrEP and PEP, risky sexual behaviors, HIV tests, and HIV knowledge. Awareness of HIV PrEP or PEP refers to having heard about HIV PrEP or PEP and having relevant knowledge of HIV PrEP or PEP, such as medication indications, drug types, administration and precautions, etc. Awareness of HIV PrEP and PEP were categorized as being “aware of PrEP,” “not aware of PrEP,” “aware of PEP,” and “not aware of PEP.” Uptake of HIV PrEP and PEP were categorized as “have sought PrEP or/and PEP,” “have not sought PrEP or PEP,” “have only sought PrEP,” and “have only sought PEP.” Pathways to learn PrEP and PEP included school clubs, the internet, the Centers for Disease Prevention and Control (CDC), hospital doctors, gay rights organizations, television programs, classmates and friends, etc. Unprotected sexual behavior refers to not ensuring the use of condoms or improper use of condoms (eg, without measures when condoms slip or break) in every sexual behavior. HIV knowledge was measured using the 8-item HIV Knowledge Questionnaire, which has been widely applied in HIV-related surveys in China and has been proven to have good validity [13]. Responses were recorded as “true,” “false,” or “don’t know” and scored as 1=“correct” and 0=“wrong or don’t know.” HIV knowledge was finally measured by the total score, with a higher score indicating a higher level of HIV knowledge.

### Statistical Analysis

Descriptive variables were used to describe sociodemographic characteristics, awareness and uptake of HIV PrEP and PEP, the distribution of pathways to learn PrEP and PEP, risky sexual behaviors, and HIV testing with frequency, percentage, and mean and SD values. Moreover, binary logistic regression analysis was used to determine the effect of factors (eg, age, gender, grade, gender of the sexual partner, sexual orientation, HIV risky sexual behavior, HIV knowledge score, and HIV testing) on seeking PrEP (ie, have sought PrEP and have not sought PrEP) and PEP (ie, have sought PEP and have not sought PEP). If the odds ratio (OR) is greater than 1, having exposure to PrEP and PEP knowledge increases the odds of having sought HIV PrEP and PEP; however, if the OR is less than 1, exposure to PrEP and PEP knowledge decreases the odds of having sought HIV PrEP and PEP. All data were analyzed using SPSS (version 25.0; IBM Corp). Variables with *P*<.05 were considered significant.

### Ethical Considerations

The study protocol and consent procedure were approved by Medical Ethics Committee of Public health school of Zhejiang University (ZGL202306-9). Participation was entirely voluntary and anonymous and based on written informed consent, and participants had the right to withdraw from the study at any time. To ensure participants’ privacy, all collected data were deidentified, and no personally identifiable information was collected or stored. The confidentiality of individuals was properly protected in the management of the investigation and the processing of data.

## Results

As of May 2023, we enrolled 21,962 college students, and 2605 college students with sexual experiences were included in our analysis.

Table 1 shows the sociodemographic characteristics of 2605 college students with sexual experiences. The average age of the participants was 21.25 (SD 2.75) years. Among them, males accounted for 62.3% (1624/2605), females accounted for 37.7% (981/2605), first-year students accounted for 33.0% (860/2605), and postgraduate students accounted for 26.5% (690/2605) of the sample. Furthermore, 58.5% (1525/2605) of college students had sex with females, and 37.4% (973/2605) of them had sex with males. Regarding sexual orientation, 88.0% (2292/2605) of students self-reported as heterosexual, while 3.9% (101/2605) of students self-reported as being homosexual.

**Table 1.** Characteristics of college students (N=2605) with sexual experiences investigated from November 2022 to May 2023.

Characteristics		Values
Age (years), mean (SD)		21.75 (2.75)
**Gender, n (%)**		
	Male	1624 (62.34)
	Female	981 (37.66)
**Grade, n (%)**		
	Freshmen	860 (33.01)
	Sophomore	509 (19.54)
	Junior	363 (13.93)
	Senior	183 (7.02)
	Postgraduate	690 (26.49)
**Gender of the sexual partner, n (%)**		
	Female	1525 (58.54)
	Male	973 (37.35)
	Both	107 (4.11)
**Sexual orientation, n (%)**		
	Heterosexual	2292 (87.98)
	Homosexual	101 (3.88)
	Bisexual	168 (6.45)
	Not sure	44 (1.69)

Table 2 indicates that compared to college students who have not sought PrEP or PEP, those who have sought PrEP or/and PEP had a higher likelihood of having more than 6 sexual partners (24/146, 16.4% vs 93/2321, 4.0%; *P*<.001), always having unprotected sex (20/146, 13.7% vs 121/2321, 5.2%; *P*<.001), having subjective perceived risk behavior (23/146, 15.8% vs 132/2321, 5.7%; *P*<.001), and undergoing an HIV test (42/146, 28.8% vs 193/2321, 8.3%; *P*<.001).

**Table 2.** Risky sexual behavior and HIV testing among college students with sexual experiences who have or have not sought PrEP^a^ and PEP^b^ investigated from November 2022 to May 2023.

		Total participants (n=2605), n (%)	Have sought PrEP or/and PEP (n=146), n (%)	Have not sought PrEP or PEP (n=2321), n (%)	Have sought only PrEP (n=94), n (%)	Have sought only PEP (n=44), n (%)	*P* value
**Number of sexual partners**							<.001
	1	2175 (83.49)	101 (69.18)	1980 (85.31)	62 (65.96)	32 (72.73)	
	2‐5	289 (11.09)	21 (14.38)	248 (10.69)	15 (15.96)	5 (11.36)	
	6	141 (5.41)	24 (16.44)	93 (4.01)	17 (18.09)	7 (15.91)	
**Ever had unprotected sex**							<.001
	Always	166 (6.37)	20 (13.70)	121 (5.21)	16 (17.02)	9 (20.45)	
	Sometime	759 (29.14)	41 (28.08)	678 (29.21)	30 (31.91)	10 (22.73)	
	Never	1680 (64.49)	85 (58.22)	1522 (65.58)	48 (51.06)	25 (56.82)	
**Ever had subjective perceived risk behavior**							<.001
	Yes	171 (6.56)	23 (15.75)	132 (5.69)	9 (9.57)	7 (15.91)	
	No	2434 (93.44)	123 (82.25)	2189 (94.31)	85 (90.43)	37 (84.09)	
**Ever undergone an HIV test**							<.001
	Yes	267 (10.25)	42 (28.77)	193 (8.32)	22 (23.40)	10 (22.73)	
	No	2338 (89.75)	104 (71.23)	2128 (91.68)	72 (76.60)	34 (77.27)	

a PrEP: preexposure prophylaxis.

b PEP: postexposure prophylaxis.

Awareness and uptake of HIV PrEP and PEP are shown in Table 3 and Multimedia Appendix 1. Overall, 61.4% (1600/2605) of the participants were aware of PrEP, and 53.0% (1380/2605) of them were aware of PEP. Furthermore, 5.6% (146/2605) of the participants have sought PrEP or/and PEP, 89.1% (2321/2605) have not sought PrEP and PEP, 3.6% (94/2605) have only sought PrEP, and 1.7% (44/2605) have only sought PEP.

**Table 3.** Awareness and uptake of HIV preexposure prophylaxis (PrEP) and postexposure prophylaxis (PEP) among college students with sexual experiences (N=2605) investigated from November 2022 to May 2023.

Parameters	Students, n (%)
Aware of PrEP	1600 (61.42)
Not aware of PrEP	1005 (38.58)
Aware of PEP	1380 (52.98)
Not aware of PEP	1225 (47.02)
Have sought PrEP and PEP	146 (5.60)
Have not sought PrEP and PEP	2321 (89.10)
Have sought only PrEP	94 (3.61)
Have sought only PEP	44 (1.69)

**Figure 1.**
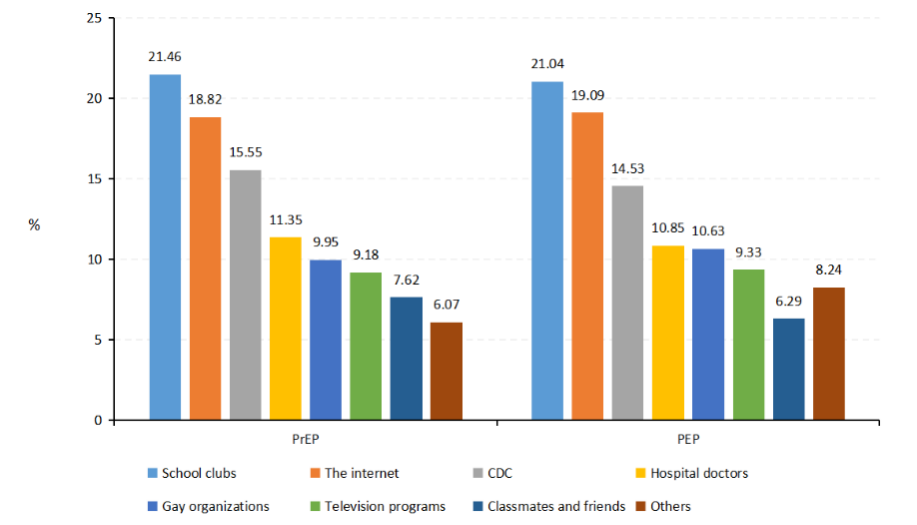
Pathways to know preexposure prophylaxis (PrEP) and postexposure prophylaxis (PEP) among college students with sexual experiences investigated from November 2022 to May 2023. CDC: Centers for Disease Prevention and Control.

Regarding pathways to learn about PrEP and PEP (Figure 1), the main ways for the participants to learn about PrEP and PEP were school clubs (n=138, 21.5% for PrEP and n=97, 21.0% for PEP), the internet (n=121, 18.8% for PrEP and n=88, 19.1% for PEP), and the CDC (n=100, 15.6% for PrEP and n=67, 14.5% for PEP).

Table 4 indicates the factors that influence seeking PrEP and PEP. Senior students and those who have not undergone an HIV test had a lower likelihood of seeking PrEP and PEP (*P*<.001). Compared with those who had sex with females only, those who had sex with both males and females (OR 2.979, *P*=.007) had a higher likelihood of seeking PrEP. Moreover, college students who did not have subjective perceived risk behavior (OR 0.468, *P*=.004) and homosexual students (OR 0.318, *P*=.03) were more likely to not seek PEP.

**Table 4.** Binary logistic regression determining the factors on seeking PrEP^a^ and PEP^b^ among college students with sexual experiences investigated from November 2022 to May 2023, after controlling for subjective perceived risk behavior, HIV knowledge score, HIV testing, age, gender, grade, gender of the sexual partner, and sexual orientation.

Items		Whether have sought PrEP				Whether have sought PEP			
		COR^c^ (95% CI)	*P* value	AOR^d^ (95% CI)	*P* value	COR (95% CI)	*P* value	AOR (95% CI)	*P* value
**Ever had subjective perceived risk behavior (reference: yes)**									
	No	0.406 (0.269‐0.611)	<.001	0.699 (0.422‐1.159)	.17	0.331 (0.216‐0.506)	<.001	0.468 (0.278‐0.786)	.004
HIV knowledge score		1.012 (0.926‐1.106)	.79	1.101 (1.000‐1.212)	.05	1.031 (0.931‐1.141)	.60	1.112 (0.997‐1.240)	.06
**Ever done HIV test (reference: yes)**									
	No	0.258 (0.187‐0.356)	<.001	0.284 (0.193‐0.417)	<.001	0.259 (0.183‐0.367)	<.001	0.271 (0.179‐0.412)	<.001
Age (years), mean (SD)		0.871 (0.825‐0.920)	<.001	0.977 (0.907‐1.052)	.54	0.866 (0.816‐0.920)	<.001	0.967 (0.891‐1.050)	.43
**Gender (reference: male)**									
	Female	0.462 (0.338‐0.631)	<.001	0.693 (0.375‐1.281)	.24	0.586 (0.421‐0.816)	.002	1.070 (0.534‐2.145)	.85
**Grade (reference: first-year students)**									
	Sophomore	0.956 (0.685‐1.333)	.79	1.018 (0.714‐1.451)	.92	1.046 (0.729‐1.501)	.81	1.101 (0.752‐1.614)	.62
	Junior	0.554 (0.356‐0.860)	.009	0.564 (0.349‐0.910)	.02	0.497 (0.298‐0.830)	.008	0.504 (0.291‐0.873)	.02
	Senior	0.441 (0.232‐0.837)	.01	0.361 (0.173‐0.753)	.007	0.411 (0.196‐0.865)	.02	0.325 (0.138‐0.763)	.01
	Postgraduate	0.324 (0.215‐0.490)	<.001	0.349 (0.199‐0.613)	<.001	0.324 (0.204‐0.516)	<.001	0.340 (0.181‐0.638)	.001
**Gender of the sexual partner (reference: female)**									
	Male	0.533 (0.388‐0.734)	<.001	0.715 (0.374‐1.365)	.31	0.608 (0.430‐0.858)	.005	0.571 (0.275‐1.185)	.13
	Both	3.631 (2.316‐5.693)	<.001	2.979 (1.351‐6.570)	.007	3.030 (1.830‐5.019)	<.001	2.103 (0.863‐5.125)	.10
**Sexual orientation (reference: heterosexual)**									
	Homosexuality	1.175 (0.602‐2.295)	.64	0.502 (0.219‐1.153)	.10	0.842 (0.363‐1.950)	.69	0.318 (0.114‐0.886)	.03
	Bisexuality	1.782 (1.129‐2.813)	.01	0.618 (0.296‐1.289)	.20	1.500 (0.886‐2.540)	.13	0.458 (0.196‐1.071)	.07
	Not sure	3.145 (1.531‐6.462)	.002	1.609 (0.636‐4.069)	.32	2.521 (1.106‐5.745)	.03	1.199 (0.415‐3.464)	.74

a PrEP: preexposure prophylaxis.

b PEP: postexposure prophylaxis.

c COR: crude odds ratio.

d AOR: adjusted odds ratio.

## Discussion

### Principal Findings

Our results show that more than half of the participants were aware of PrEP and PEP, but they rarely sought PrEP and PEP. In total, 61.4% (1600/2605) of the participants were aware of PrEP, which was much higher than the proportion of college students in other provinces (13.9%) [14]. This finding may be related to different surveyed areas, where our study site has continuously strengthened HIV prevention among young students in recent years. The proportion of HIV awareness among young students in the latest year increased from 82.3% in 2016 to 96.2% in 2021 [15], which may be related to the participants’ awareness of PrEP. In addition, 53.0% (1380/2605) of the participants were aware of PEP, which was slightly lower than the proportion reported in a survey conducted in India targeting medical college students (57.94%) [16]. This may be because medical students might have better knowledge of reproductive health including PEP. However, in general, it is still concerning that almost half of college students had insufficient awareness of PrEP and/or PEP, which indicates that they may have limited access to key prevention drugs since they were not aware that PrEP or PEP existed and were unable to take measures to prevent HIV infection before or after risk exposure, which may result in serious consequences [17,18].

Only 5.6% of the participants have sought PrEP or/and PEP. Colleges in China have been paying more attention to condom placement, increasing HIV test sites, and HIV counseling to promote HIV prevention among young students [19]. There are fewer campaigns about PrEP and PEP aimed at students, which may have resulted in a relatively low seeking rate, and it may have an important impact on HIV infection after risk exposure [20]. Therefore, seeking of PrEP and PEP is particularly important for college students with sexual experiences, especially with high risk exposure. Due to the concentration and relatively closed features of campus life, once students contract the virus, they could easily transmit it to their sexual partner, even spreading it widely on campus. In fact, this should be an avoidable behavior, as seeking PEP timely after exposure could effectively prevent HIV infection [21].

The main ways to learn about PrEP and PEP were school clubs and the internet. A 2-year HIV health education intervention study reported that delivering HIV health education in colleges through student communities could effectively improve the HIV/AIDS-related knowledge, attitudes, and behaviors [22]. Because of the widespread popularity of smartphones, the accessibility of the internet has increased, and mass media and the internet have become increasingly popular sources of health information [23]. A cross-sectional survey suggested that adolescents found informal sources more useful and experienced higher levels of comfort accessing informal support [24]. In addition, a small number of the participants gained relevant knowledge from gay rights organizations. Considering the attribute of education in school settings, it is more appropriate for professional institutions such as specialist education organizations in college and the CDC to intervene rather than propaganda spread by gay rights organizations.

Compared with college students who have not sought PrEP or PEP, those who have sought PrEP or/and PEP had a higher proportion of having risky sexual behaviors. After taking PrEP drugs, they may think they cannot be infected with HIV or have a reduced probability of HIV infection as long as they took PrEP drugs, which may promote risky sexual behaviors. Moreover, college students who have undergone HIV testing are more likely to seek PrEP and PEP. A higher acceptability of PrEP and PEP was also reportedly associated with having had a prior HIV test [25]. Considering the potential risk exposure underlying an HIV test, it is reasonable that the more HIV tests people do, the greater the likelihood of using PEP [26]. Compared with heterosexual students, homosexual students had a lower likelihood of seeking PrEP and PEP. Homosexual students were reportedly more prone to exhibit risky sexual behaviors [27], but the risk of HIV infection might not directly prompt them to take preventive measures. One of the main reasons may be that homosexual students underestimated their risk of HIV infection, which affected their judgment about whether they need to use PrEP or PEP, forming a behavior-cognition bias [28]. Therefore, more efforts should be made to assist homosexual individuals in assessing risks appropriately, especially for men who have sex with men who meet the World Health Organization’s criteria for PrEP and PEP use.

PrEP and PEP drugs are also used for HIV treatment, and since homosexual individuals may be afraid of being mistaken for having HIV, they may have preferred not to use the drugs due to stigma [29]. Peer education to promote PrEP and PEP is helpful to reduce their fear of discrimination.

Some researchers believe that self-perceived low efficacy, concern about side effects, adherence, and affordability are the main barriers for homosexual individuals to use PrEP and PEP, and robust awareness of PrEP and PEP helps promote the willingness to take them [30,31]. These studies suggest that provision of comprehensive and accurate information about efficacy, safety, and preferential policies of PrEP and PEP drugs to men who have sex with men are essential to allay their concerns.

### Limitations

Our study has several limitations. First, the sample for data analysis is college students with sexual experiences, but we could not identify the frequency of sexual behaviors and the sources of their sexual partners, which limit in-depth analysis. Second, considering the social desirability of sexual behaviors, the participants may respond to the questions with answers that meet social expectations. Moreover, owing to significant variations in cultures, the economy, and traditions across China, data from one province are unlikely to represent the whole population of college students nationwide.

### Conclusion

College students with sexual experiences rarely seek PrEP and PEP and have a relatively low awareness of PrEP and PEP. It is necessary to promote the awareness of PrEP and PEP services for this group of people who are at risk of HIV infection, considering that PEP facilitates HIV prevention.

## Supplementary material

10.2196/63211 Multimedia Appendix 1 Graphical representation of the awareness and uptake of HIV pre-exposure prophylaxis (PrEP) and postexposure prophylaxis (PEP) among college students with sexual experiences (N=2605) investigated from November 2022 to May 2023.

## Abbreviations

CDC: Centers for Disease Prevention and Control
OR: odds ratio
PEP: postexposure prophylaxis
PrEP: preexposure prophylaxis
UNAIDS: Joint United Nations Programme on AIDS
