# Supplementary figures and images for: Awareness and Uptake of HIV Preexposure Prophylaxis and Postexposure Prophylaxis Among College Students With Sexual Experiences: Institutional-Based Cross-Sectional Study
Source: JMIR Public Health Surveill. 2024 Nov 6;10:e63211. doi: 10.2196/63211 (PMC11560078; doi:10.2196/63211)

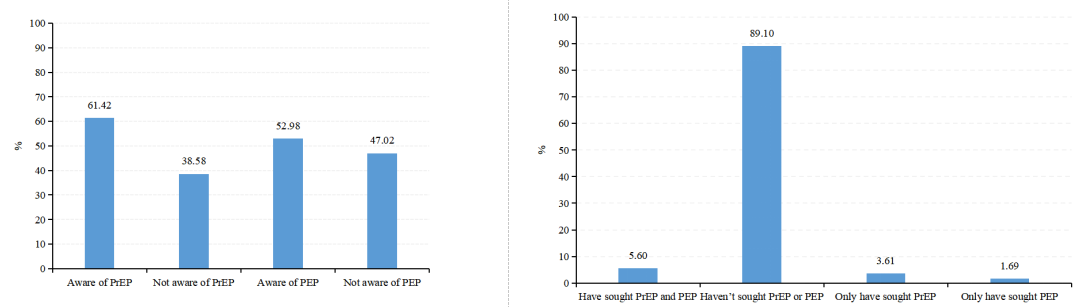

Supplement: Multimedia Appendix 1 [file publichealth-v10-e63211-s001.png]
